# Patterns of bruising in preschool children—a longitudinal study

**DOI:** 10.1136/archdischild-2014-307120

**Published:** 2015-01-14

**Authors:** Alison M Kemp, Frank Dunstan, Diane Nuttall, M Hamilton, Peter Collins, Sabine Maguire

**Affiliations:** 1 Early Years Research Programme, Institute of Primary Care and Public Health, School of Medicine, Cardiff University, Cardiff, UK; 2 Arthur Bloom Haemophilia Centre, Institute of Infection and Immunity, School of Medicine, Cardiff University, Cardiff, UK

**Keywords:** Child Abuse, Injury Prevention

## Abstract

**Introduction:**

This study aims to identify the prevalence and pattern of bruises in preschool children over time, and explore influential variables

**Methods:**

Prospective longitudinal study of children (<6 years) where bruises were recorded on a body chart, weekly for up to 12 weeks. The number and location of bruises were analysed according to development. Longitudinal analysis was performed using multilevel modelling.

**Results:**

3523 bruises recorded from 2570 data collections from 328 children (mean age 19 months); 6.7% of 1010 collections from premobile children had at least one bruise (2.2% of babies who could not roll over and 9.8% in those who could), compared with 45.6% of 478 early mobile and 78.8% of 1082 walking child collections. The most common site affected in all groups was below the knees, followed by ‘facial T’ and head in premobile and early mobile. The ears, neck, buttocks, genitalia and hands were rarely bruised (<1% of all collections). None of gender, season or the level of social deprivation significantly influenced bruising patterns, although having a sibling increased the mean number of bruises. There was considerable variation in the number of bruises recorded between different children which increased with developmental stage and was greater than the variation between numbers of bruises in collections from the same child over time.

**Conclusions:**

These data should help clinicians understand the patterns of ‘everyday bruising’ and recognise children who have an unusual numbers or distribution of bruises who may need assessment for physical abuse or bleeding disorders.

**What is already known on this topic**

- A handful of published studies show bruising is unusual in non-mobile infants and increases as children become more mobile.
- The prevalence of bruises at different sites with respect to development is ill- defined as is the proportion of children who bruise more frequently than others.

**What this study adds**

- Bruising affects a small proportion of babies who cannot roll over.
- Rare sites for bruising: ears, neck, genitalia, hands, in any child and buttocks and front trunk in early and premobile children.
- Nine per cent of children have twice as many bruises as would be expected for their developmental stage.

## Introduction

Paediatricians faced with a child with concerning bruising must distinguish between accidental bruising, physical child abuse and congenital or acquired bleeding disorders. They must therefore understand the patterns of bruising in children from day-to-day activities, and any influential factors.

Studies show that bruising changes as children become increasingly mobile^1–3^ and is unusual in infants who are not yet mobile.^3^ However there is a small literature base defining ‘normal’ patterns of bruising.^4^ ^5^ Accidental bruises are most likely to appear over the bony prominences, such as knees and shins, and on the front of the body.^4^ Chang described an occipital and ‘facial T’ distribution of bruises in children experiencing slips, trips and falls.^6^ Previous studies have used selected populations, such as children attending baby clinics and outpatient appointments,^1–3^ ^7^ children who had other injuries, such as fractures,^8^ or children admitted to paediatric intensive care with head injuries.^9^ These studies give limited insight into ‘day-to-day’ patterns of bruising in children.

Factors proposed to affect the extent of bruising include the child's stage of development, family order, season and gender.^2^ Many parents report that their child ‘bruises easily’, but no studies have explored child-to-child variation nor the extent to which the number of bruises in a given child varies over time, with the exception of one small report of six children (3–4 years old) in a playgroup with weekly observations over 6 weeks, that recorded at least three bruises in every collection.^10^

This study aims to describe the number and distribution of bruises sustained from everyday activities and accidents in a UK population of preschool children, to explore between and within child variation, the changing pattern of bruises with stages of motor development, and the relationship between bruising and gender, ethnicity, season, sibling order and socioeconomic status.

## Methods

This prospective longitudinal study recruited children (age 0–6 years) from South Wales, UK. Parents were recruited from well-baby clinics, hospital outpatient clinics, and mother and baby groups in the local community. Children with clinically documented motor disability, confirmed bleeding disorders or suspected child abuse were excluded. Those with a family history of a hereditary bleeding disorder were included if coagulation testing had excluded the condition.

The study had two phases: Phase 1 (April 2005–December 2007) and Phase 2 (April 2008–August 2011). The latter followed an amendment to the ethical approval that allowed us to collect additional data-fields. In phases 1 and 2, data were collected on gender, age, developmental stage and past medical history. In phase 2 we added ethnicity, family order and socioeconomic status based on the Townsend score^11^ and parents were asked to record the cause of the bruise when known. The postcode of the child's residence was mapped into the Lower Super Output Area (LSOA). Townsend deprivation scores have been calculated for all LSOAs, and grouped into five quintiles based on this score. The quintile of deprivation was taken as an ecological measure of deprivation for a child (5th quintile representing the greatest level of deprivation). Parents were trained to recognise a bruise as a non-blanching red/blue/purple mark that appeared on the skin, and record the number and location of bruises in their own home. They were trained not to record cuts, abrasions, birthmarks or other skin lesions. Bruises were recorded on a body map at a weekly data *collection* for up to 12 weeks. Once children were walking, four weekly collections were accepted.

At each data collection parents recorded the most advanced motor developmental milestone achieved. Children were classified as premobile (subdivided into baby not yet rolling over and those able to roll over), early mobile (crawling or cruising) and walking. Some children were included for a subsequent 12 week period if they had progressed to a different developmental stage. Data from the two phases were combined for analysis where possible.

A random sample of 40 data collections from 40 different children was selected to validate the quality and consistency of recording. The parent recorded the number, location and size of bruises (maximum diameter), and a single research nurse verified that the lesions recorded were bruises by independently repeating this process on the same day. The parents were not warned that the nurse would visit on the day to validate the data collection.

**Figure 1:**
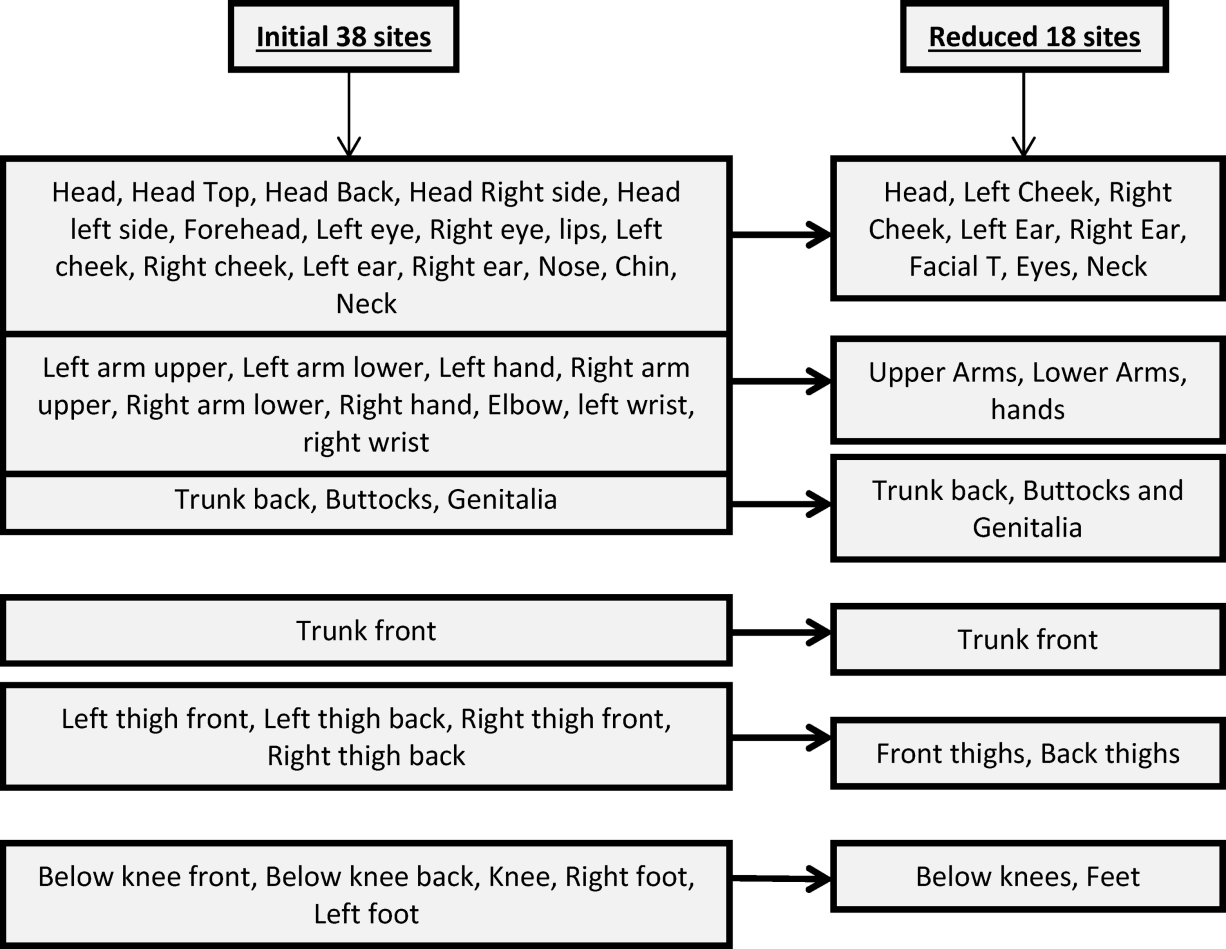
Categorisation of sites where the locations of bruises were recorded at each data collection.

Bruises were recorded in 38 different locations on the body. These were grouped into 18 after an initial analysis, combining contralateral locations where laterality was unimportant or where bruises were rare, for example, wrists (figure 1). The ‘facial T’ was defined as the forehead, nose, mouth (upper or lower lip) or chin.^6^ The ‘head’ refers to the area within the hairline. Bruises from immunisations or venipuncture were excluded. All data were entered onto an Access database using a process of double data entry.

## Statistical analysis

Data were summarised using mean and SD, for continuous or count data, and proportions for binary data. The date of each collection was coded into a seasonal variable with four categories (table 3). Longitudinal analysis was performed on the number of bruises using multilevel modelling with Poisson distribution and a log link function.^12^ Developmental stage, gender, ethnicity, socioeconomic status, the presence of siblings and seasonality were added to the model to assess their importance.^13^

## Results

One thousand and two parents were approached to participate, 380 gave informed consent and 328 children (mean age 19 months, 54% female) had at least one collection, providing a total of 2570 data collections (590 collections from 52 children were from phase 1), with an average of 7.8 collections per child. Forty-two per cent provided 12 *collections* and 39% between 4 and 11, with three per cent providing more than 12 collections spread over more than one development stage. Forty children had collections spanning the premobile and early mobile phases and 27 spanned early mobile and walking phases. Ethnicity was recorded in 280 children; 269 were white British and 11 were in other ethnic groups (similar to the ethnic mix of the local population). The Townsend quintile was available for 245 children; 46% were from the least and 15% from the two most deprived quintiles. At recruitment 133 (41%) children were premobile, 43 (13%) early mobile and 152 (46%) walking. Some children changed developmental stage during data collection; 39% of the 2570 *collections* were in premobile children, 19% in early mobile and 42% in walkers. The validation process showed complete agreement between parents and the research nurse for the number and site of bruise recordings. For bruise size (within 5 mm) agreement was 55% (44/80); size is not included in this analysis.

A total of 3523 bruises were recorded across all collections. The percentage of collections with at least one bruise, the mean number of bruises per collection, and the mean number of sites affected increased with incremental development stage (table 1). Because of multiple collections in a child, this table does not show the prevalence of bruising in children. The prevalence of bruising in the first collection for each child was 5.3% in premobile, 55.8% in early mobile and 87.5% in walking children.

**Table 1:** Number and percentage of 2570 collections with at least one bruise, mean number (and range) of bruises and sites affected, with SDs, by developmental stage

	Number and percentage with 95% CI of collections with at least one bruise	Mean (SD) number of bruises per collection	Mean (SD) number of sites affected per collection	Range, median and 90th centile of number of bruises per collection
Premobile Age range 0–11 months	68/1010 6.7% (95% CI 5.3 to 8.4)	0.09 (0.35)	0.08 (0.30)	0–3 0 0
Early mobile Age range 4–18 months	218/478 45.6% (95% CI 41.2 to 50.1)	0.80 (1.19)	0.59 (0.79)	0–7 0 2
Walking Age range 10–70 months	852/1082 78.8% (95% CI 76.2 to 81.1)	2.82 (2.77)	1.50 (1.23)	0–16
10–36 months	422/594 71.0% (95% CI 67.3 to 74.5)	2.31 (2.59)	1.28 (1.17)	0–14 2 6
37–70 months	387/430 90.0% (95% CI 86.8 to 92.4)			0–16 3 7
Overall Age range 0–70 months	1138/2570 44.8% (95% CI 42.4 to 46.2)	1.37 (2.27)	0.77 (1.10)	0–16 0 4

Twelve bruises were recorded in 9 of 405 collections (2.2%, 95% CI 1.2% to 4.2%) in children who were not yet able to roll over. The cause, when reported, included bumping into mother's tooth, falling asleep on a dummy, banging themselves with a fist or rattle and a toy that was dropped on one baby. There were 75 bruises in 59 of 605 collections (9.8%, 95% CI 7.6% to 12.4%), in children who could roll over but were not yet crawling. Examples of causes included 12 children who had fallen or toppled over, 7 rolled into something, 4 banged into an object and 6 hit themselves with an object.

**Figure 2:**
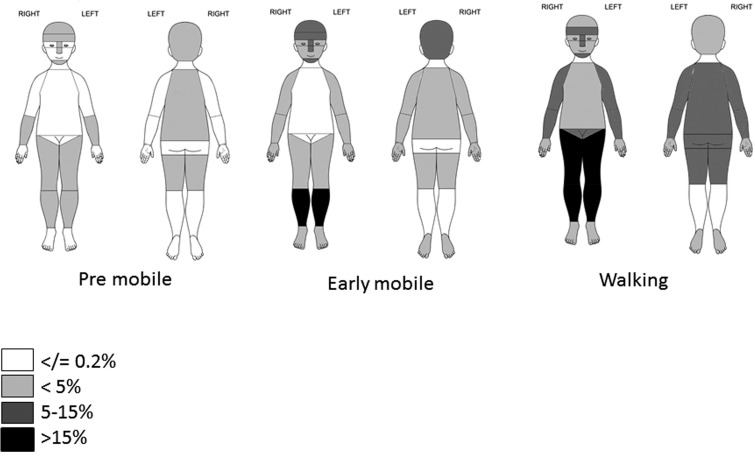
Distribution of percentage of 2570 collections from 328 children with at least one bruise by location and development stage.

The most common sites for bruises in premobile and early mobile children were below the knees, the ‘facial T’ and head (table 2 and figure 2). In the walking group the most common site was below the knees, although many other locations were affected, notably the front of thighs, lower arms and rear trunk (where 78% of bruises were to the lower back). Some sites had numerous bruises. The mean number of bruises below the knee was 2.8 and to the front of the thigh was 1.4 in collections where at least 1 bruise was present. The ears, neck, genitalia and hands were rarely bruised (<1% of *collections*) in any developmental group. Buttocks and front trunk were rare sites (<0.2%) in premobile and early mobile children. When bruises to the front trunk were reported they occurred in children who were walking and 95% were over the bony prominences of the iliac crest, clavicle, ribs or shoulder.

**Table 2:** Number and percentage of 2570 collections from 328 children in which there was at least one bruise by location and development stage; the numbers of collections by locations do not sum to the total for all bruises as there may be more than one location affected in a single location

	Premobile 1010 collections		Early mobile 478 collections		Walking 1082 collections	
	Number of collections	Per cent	Number of collections	Per cent	Number of collections	Per cent
Left cheek	1	0.1	11	2.3	15	1.4
Right cheek	1	0.1	12	2.5	18	1.7
Left ear	0	0.0	1	0.2	0	0.0
Right ear	0	0.0	1	0.2	1	0.1
Head	12	1.2	32	6.7	34	3.1
Facial T	13	1.3	65	13.6	122	11.3
Eyes	0	0.0	8	1.7	12	1.1
Front trunk	2	0.2	0	0.0	43	4.0
Rear trunk	9	0.9	11	2.3	104	9.6
Neck	0	0.0	1	0.2	0	0.0
Buttocks	0	0.0	1	0.2	54	5.0
Genitalia	0	0.0	0	0.0	0	0
Upper arms	2	0.2	6	1.3	99	9.1
Hands	2	0.2	3	0.6	6	0.6
Lower arms	5	0.5	6	1.3	126	11.6
Front thighs	10	1.0	18	3.8	181	16.7
Back thighs	4	0.4	3	0.6	72	6.7
Feet	2	0.2	6	1.3	45	4.2
Below knees	14	1.4	99	20.7	693	64.0
All bruises	68	6.7	218	45.6	852	78.7

To assess the variation between children, the data were separated into different developmental stages. The majority of collections in the premobile children had no bruises and 73% (97/133) of children never had a bruise recorded over 1010 collections. For those with independent mobility, there was wide variation between children and for many children there was considerable variation between collections. For walking children the percentage of the total variation due to differences between children was 75.4%; for early mobile and premobile children the corresponding values were 60.5 and 41.8. For walking children, the majority of the variation was between children, suggesting that different children tend to sustain different amounts of bruising, but there was substantial variation between children for all developmental groups.

For this reason a multilevel analysis was performed to analyse the data on collections nested within children. Fitting this model to the 1980 collections from phase 2, the estimated ratio, comparing the mean number of bruises in children walking with early mobile children, was 2.35 (95% CI 1.95 to 2.84). This means that a child who is walking would be expected to have more than twice as many bruises on average as an early mobile child. The ratio in premobile children compared with early mobile was 0.07 (95% CI 0.05 to 0.09).

Table 3 shows estimates of these effects adjusted for: gender, seasonality, ethnicity, deprivation and the presence of siblings. These estimates are multiplicative effects, in that the risk ratios above are multiplied by these terms. Having a sibling significantly increased the mean number of bruises (the sibling order had no effect); season, ethnicity and gender effects were not significant. Although bruising was more extensive in the most deprived quintile, this result was not statistically significant. There was considerable variation in the amount of bruising a child displayed, even when adjusting for developmental stage and the above factors. More than nine per cent of children had more than twice the expected amount of bruising while five per cent had fewer than half the expected number of bruises.

**Table 3:** Associations between the number of bruises and developmental stage and other child-level factors; the ratios of means, and the associated 95% CIs, between the category of the factor and the reference category

Factor	Ratio of means	95% CIs
Development stage		
Premobile	0.06	0.04 to 0.09
Early mobile (reference)	1	
Walking	2.28	1.73 to 2.99
Gender (female)	0.84	0.64 to 1.09
Any sibling	1.51	1.15 to 1.99
Season		
January–March (reference)	1	
April–June	0.99	0.82 to 1.20
July–September	0.94	0.74 to 1.18
October–December	0.82	0.63 to 1.06
Ethnicity		
White	1	
Ethnicity (not White)	0.90	0.46 to 1.79
Not recorded	0.84	0.58 to 1.23
Deprivation		
Quintile 1	1	
Quintile 2	0.96	0.70 to 1.32
Quintile 3	1.14	0.79 to 1.65
Quintile 4	0.94	0.59 to 1.48
Quintile 5	1.56	0.91 to 2.66

## Discussion

This large longitudinal study confirms a strong relationship between the presence, number and location of bruises and different stages of motor development. Bruising in babies who were not yet rolling over was uncommon. The percentage of collections with at least one bruise, and the number of bruises at each collection, increased with incremental developmental stage. The most common sites of bruising were below the knees, ‘facial T’ and head, while rare sites included the ears, neck, genitalia, hands, in any child and buttocks and front trunk in early mobile and premobile children.

There are few studies of bruising in young children. A longitudinal cohort study explored parental reports of injuries in 11 466 children, younger than 6 months of age. Parents recorded 3357 falls in 22% (2554) of these children, visible injuries were reported in 14% of these cases, 56% of which were bruises. Most visible injuries (97%) were to the head. The study concluded that accidents were surprisingly common in infants aged 6 months and younger. The majority were falls, however, injuries were infrequent, and generally trivial.^14^

Other studies have shown that older children have more bruises than younger children.^1–3^ ^15–17^ and two studies showed that bruising increased with developmental stage.^1^ ^3^ For all developmental groups the prevalence of bruises in this study was greater than in previous studies (table 4). Explanations for this may include the method of data collection; parents may have been more vigilant in recording faint bruises that they had been monitoring for days prior to the collection date. It is possible that bruises that had not resolved over the weekly interval between collections may have been double counted. However the mean numbers of bruises were similar in the first collection for each child to those for later collections.

**Table 4:** Prevalence and number of bruises at different development stages and ages as recorded in previous studies; the data from the current study refer to the first collection only for the child, to make them comparable with the other studies

	Percentage of children with bruises (95% CI) and number of bruises per developmental stage			
	Sugar *et al*^3^	Carpenter^1^	Labbe and Caouette^2^	Kemp *et al* (per collection)
Development stage	Precruisers 2.2 (1.2 to 5.8)	Precrawlers:3.9 (1.6 to 9.7)		Premobile:not rolling over, 1.3 (0.2 to 6.9)
				Rolling over 10.9 (5.1 to 21.8)
	Cruisers 17.8 (11.6 to 26.4))	Crawlers 17.3 (9.4 to 29.7)		Early mobile:55.8 (41.1 to 59.6)
	Walkers 51.9 (46.4 to 57.3)	Walkers 37.5 (21.2 to 57.3)		Walkers 87.5 (81.3 to 91.8)
Age in months	<6:0.5 (0.1 to 2)		<8:1.2% (95% CI 0.4 to 3.5)	
	6–12:10.6 (7.2 to 15.3)	6–12:12.4% (8.3 to 18.1)		
	12–24:42 (35.4 to 38.3)			
	24–35:60.9 (51.7 to 69.3)		9–48:60.3 (57.2 to 63.2)	
Mean number of bruises in children who had a bruise (range)	Precruisers 1.3 (range 1–2			Premobile 1.1 (1–2)
	Cruisers range (1–5)			Early mobile 2.0 (1–6)
	Walkers:2.4 (range 1–11)			Walking 3.5 (1–13)

Any bruise in premobile children raises the suspicion of physical abuse and while this could not be firmly excluded in every case, the probability of abuse in this population was low. The explanations given for the bruises, where available, were compatible with the bruise sustained. In the few cases where bruise pattern was deemed unusual, they were independently reviewed by a child protection team and abuse was excluded.

There was considerable variation in the number of bruises observed between different children at the same developmental stage, especially at more advanced developmental stages. There was also considerable variation within the same child at different time points. Nine per cent of children had more than twice the expected number of bruises over repeated collections. Labbe and Caouette^2^ also noted that there were a few children who had far more bruises than the average for that age group. This is not necessarily a reflection of a child who ‘bruises easily’; other explanations include high activity levels. It is also possible that some children within the general population may have an undiagnosed mild bleeding disorder, although these disorders are rare, affecting less than 0.1% of the UK population.^18^

There was no gender or seasonal variation contrary to the Canadian study.^2^ Seasonal weather patterns vary more dramatically in Canada than in the UK, and may affect children's lifestyles to a greater extent. The significant influence on the number of bruises sustained when a child was part of a sibling group may reflect lower levels of individual supervision or the effects of sibling play.

The proportion of collections with below knee bruises increased with motor development, while head and ‘facial T’ bruises peaked in the early mobile group. Bruises became more widely distributed in the walkers. Sugar *et al* identified very similar distributions in the three equivalent developmental groups.^3^ Two smaller studies^7^ ^17^ identified the high prevalence of below knee bruises, followed by the broad category of ‘head bruises’ in early mobile and walking children. Chang *et al* analysed craniofacial injuries and described a peak incidence of trips and slips in toddlers, and falls in those younger than 1 year.^6^ The most common site of bruising was the ‘facial T’, followed by the back of the head. These injury mechanisms may explain this distribution across the developmental groups.

Bruises on the ears, neck, genitalia and hands were rare in any developmental group and buttock and front trunk bruises were rare in early mobile and younger children. A strength of the data set is that it included the anogenital area, excluded by previous authors.^2^ Sugar *et al* identified buttocks/hands/feet/abdomen/ upper arms as rare sites but did not mention ears or neck.^3^ Pierce *et al* detailed data on a highly selected group of 53 children younger than 48 months admitted to the pediatric intensive care unit with accidental trauma.^9^ Bruises were identified in 38 children, a similar profile of rare sites for accidental bruising was identified.

Examples of causes of bruises were given, particularly in the premobile and early mobile children. As missing data in this field may represent either no knowledge of the cause, or a failure to record the information, we could not interpret how frequently parents know the cause of each bruise.

This study design has the benefit of prospective data collection using a consistent approach. The study relied upon the co-operation of 328 parents to collect data. The less socially deprived population was over-represented in our sample, possibly reflecting parental willingness to comply with the study, but this should not bias the associations with bruising. Study compliance varied in terms of the length of time each participant was involved, and while we cannot be certain that every single bruise was collected we have a high degree of confidence in our participants, verified by our validation of data collection accuracy. Recruitment to the study was challenging as it required prolonged participation. The informed consent described the reasoning behind the study, including a need to identify normal patterns of bruising to compare with bruising in child abuse. Parents were informed that a referral to social services would be made if child maltreatment was suspected. This information tended to dichotomise our target audience so that parents were either highly motivated to participate or vehemently declined. This may have impacted on willingness to participate. Each collection was scrutinised and at no time was a suspicion of abuse raised and no child was referred to social services during the study time period.

These data have the potential to alert clinicians to an unusual number or distribution of bruises in young children. Multiple bruises in premobile babies and bruises in locations other than the ‘facial T’, head or below the knee would be an unusual finding as would bruises to the ear, neck, hands or genitalia. Thus these data create invaluable baseline information for those assessing bruises in children.
